# Enlargement of Cerebral Ventricles as an Early Indicator of Encephalomyelitis

**DOI:** 10.1371/journal.pone.0072841

**Published:** 2013-08-22

**Authors:** Stefano Lepore, Helmar Waiczies, Jan Hentschel, Yiyi Ji, Julia Skodowski, Andreas Pohlmann, Jason M. Millward, Friedemann Paul, Jens Wuerfel, Thoralf Niendorf, Sonia Waiczies

**Affiliations:** 1 Berlin Ultrahigh Field Facility (B.U.F.F.), Max Delbrueck Center for Molecular Medicine, Berlin, Germany; 2 Experimental and Clinical Research Center (ECRC), a joint cooperation between the Charité Universitätsmedizin Berlin and the Max Delbrueck Center for Molecular Medicine, Berlin, Germany; 3 Institute for Medical Immunology, Charité Universitätsmedizin Berlin, Berlin, Germany; 4 NeuroCure Clinical Research Center, Charité Universitätsmedizin Berlin, Berlin, Germany; 5 Institute of Neuroradiology, University Medicine, Göttingen, Göttingen, Germany; Washington University, United States of America

## Abstract

Inflammatory disorders of the central nervous system such as multiple sclerosis and acute disseminated encephalomyelitis involve an invasion of immune cells that ultimately leads to white matter demyelination, neurodegeneration and development of neurological symptoms. A clinical diagnosis is often made when neurodegenerative processes are already ongoing. In an attempt to seek early indicators of disease, we studied the temporal and spatial distribution of brain modifications in experimental autoimmune encephalomyelitis (EAE). In a thorough magnetic resonance imaging study performed with EAE mice, we observed significant enlargement of the ventricles prior to disease clinical manifestation and an increase in free water content within the cerebrospinal fluid as demonstrated by changes in T_2_ relaxation times. The increase in ventricle size was seen in the lateral, third and fourth ventricles. In some EAE mice the ventricle size started returning to normal values during disease remission. In parallel to this macroscopic phenomenon, we studied the temporal evolution of microscopic lesions commonly observed in the cerebellum also starting prior to disease onset. Our data suggest that changes in ventricle size during the early stages of brain inflammation could be an early indicator of the events preceding neurological disease and warrant further exploration in preclinical and clinical studies.

## Introduction

Under normal physiological conditions, immunologically competent CNS resident cells, such as microglia, or immune cells derived from the peripheral circulation serve to protect the CNS against potentially harmful infectious agents or traumatic events [1–3]. Blood-borne immune cells, including memory T cells, are limited to the leptomeningeal, perivascular and ventricular cerebrospinal fluid (CSF) spaces [2,3]. In the healthy brain, the CSF compartment appears to be the only site where memory T cells localize to in the CNS [4], their presence persisting for several years [3]. The CSF compartment indeed assumes an important role for immune surveillance [5,6] as the site where activated T cells can communicate with resident antigen-presenting cells [7,8]. The passage of CD4+ memory T cells into the CSF compartment occurs following their extravasation via microvascular structures of the choroid plexus into the cerebral ventricles or alternatively via postcapillary venules into leptomeningeal and perivascular Virchow–Robin (VRS) spaces [5].

Although CD4+ memory T cells can easily enter the CSF compartment, their entry into the CNS parenchyma is restricted by the blood brain barrier (BBB), which consists of the cerebral vessel endothelial cells and surrounding glia limitans. During autoimmune encephalomyelitis, such as occurs in multiple sclerosis (MS), the BBB is transformed and its function altered, e.g. by the action of several inflammatory molecules [9,10]. Following activation of the BBB, T cells now gain access to the brain parenchyma via a multi-step process that involves crossing both, the vascular endothelium and the glia limitans [11]. This inflammatory cell invasion into the VRS precedes perivascular cuffing that ultimately leads to inflammatory and demyelinating lesions. These prominent pathological patterns have long been documented histologically in MS autopsy material [12]. Specific pathological changes such as enlargement of the VRS, can be detected in MS brains with magnetic resonance imaging (MRI) [13], making it possible to detect [14] and quantify [15] these changes during the development of MS disease.

Recently, cryogenically-cooled ^1^H coils have started gaining momentum in preclinical microscopic (micro) MR neuro- and cardiovascular imaging due to an improved signal-to-noise ratio compared to traditional room temperature ^1^H coils [16–18]. Using the experimental model of autoimmune encephalomyelitis (EAE) and a cryogenically-cooled ^1^H coil, we recently were able to detect and follow pathological changes in the CNS prior to onset of clinically detectable disease signs [19]. In this study we were able to define changes in the brain of immunized mice that corresponded to cellular infiltrates in immunohistology. Moreover, after Gd-DTPA administration we observed that contrast agent (CA) distributed not only into lesions but also into the ventricular compartment in most EAE animals [19].

Here, we employed the cryogenically-cooled ^1^H coil to identify both micro- and macroscopic changes prior to clinical onset and during manifestation of disease in EAE using a larger cohort of animals. To study microscopic changes, we focused on the evolution of cerebellar lesions, which are the most common pre-symptomatic lesions in the EAE. The main macroscopic alteration in the EAE brain is a prominent and sustained increase in cerebral ventricle size. We therefore followed up changes in ventricle volume as well as T_2_ relaxation times of ventricular CSF (since this has been shown to correlate with methods measuring direct water concentration in the brain [20,21]) prior to and after clinical disease onset.

## Material and Methods

### Induction of EAE

Female SJL/J mice were purchased from Janvier (Janvier SAS, Le Genest-St-Isle, France). For active EAE, 22 mice (12 weeks old) were immunized subcutaneously with 250 µg PLP (Pepceuticals Ltd., UK) and 800 µg mycobacterium tuberculosis H37Ra (Difco) in 200 µl emulsion containing equal volumes of phosphate/buffered saline (PBS) and complete Freunds adjuvant (BD-Difco), as previously described [22] 200 ng pertussis toxin (List Biological Laboratories, US) were administered intraperitoneally at days 0 and 2. Six mice were used as normal controls. An additional 6 mice were used for sham immunizations; for this we employed the same protocol used for EAE induction but omitted PLP. Mice were weighed and scored daily as follows: 0, no disease; 1, tail weakness and righting reflex weakness; 2, paraparesis; 3, paraplegia; 4, paraplegia with forelimb weakness or paralysis; 5, moribund or dead animal. Mice with a score of 2.5 or more received an intraperitoneal injection of 200 µl glucose (5%) daily and mice with a score of 3 for more than 24 hours were sacrificed. Animals used as controls were healthy untreated mice. Animal experiments were carried out in accordance with the guidelines provided and approved by the Animal Welfare Department of the LAGeSo State Office of Health and Social Affairs Berlin (Permit G-0172/10: MR Bildgebung d. Therapieansätze - EAE 09.2010 - 09.2013).

### Magnetic Resonance Imaging

Mice were imaged before EAE immunization and daily (at the same time of day) between 5 and 18 days after active EAE induction. MRI was performed using a 9.4 Tesla animal scanner (Biospec 94/20 USR, Bruker Biospin, Ettlingen, Germany) and a cryogenically-cooled quadrature-resonator (CryoProbe, Bruker Biospin, Ettlingen, Germany). Mice were placed on a water circulated heated holder to ensure constant body temperature of 37 °C and kept anesthetized using a mixture of isoflurane 1–1.5% (Abbott GmbH & Co. KG, Wiesbaden, Germany), air and oxygen. Body temperature and breathing rate were constantly checked by a remote monitoring system (Model 1025, SA instruments Inc., New York, USA).

Horizontal and coronal fat suppressed turbo spin echo T_2_-weighted TurboRARE (TE = 36 ms, TR = 3000 ms, matrix = 384 × 384, FOV = 1.8 cm, NEX = 2, coronal slices = 22, slice thickness = 0.5 mm, scan time = 5 min, axial slices = 16, slice thickness = 400 µm, scan time = 5 min) brain images were acquired. Slice positioning was kept fixed through the longitudinal brain examination: horizontal slices were positioned parallel to the base of the brain, coronal slices were positioned perpendicularly to horizontal slices and covering the brain from the olfactory bulb/frontal lobe fissure to the cervical spinal cord. For parametric mapping of the relaxation time T_2_ a multislice-multi-echo (MSME) technique (TR = 1500 ms, matrix = 256x 256, FOV = 1.8 cm, NEX = 2, slice = 1, scan time = 15 min) was used. For this purpose echo times (TE) ranging from (10–80) ms were used to vary T_2_-weighting in increments of 10 ms. The slice was positioned horizontally using as reference the horizontal TurboRARE geometry covering cortex, caudate putamen, hippocampus, cerebellum and lateral, third and fourth ventricles.

### Brain Segmentation

Quantification of ventricle size was performed using FSL5.0 (FMRIB’s Software Library, www.fmrib.ox.ac.uk/fsl) [23–25]. All T_2_-weighted images were corrected for bias field inhomogeneity and cleared of non-brain tissue using the brain extraction tool (BET) of FSL [26]. One brain image set was chosen as reference and processed using an automated image segmentation tool (FAST) to obtain a ventricle mask [27]. Subsequently all other brain images were registered to the reference brain using FMRIB’s Linear Image Registration Tool (FLIRT) to generate an inverse transformation matrix [28]. This matrix was afterwards applied to the reference ventricles mask to map the ventricle size of each animal. To investigate volume changes in each ventricle separately a mask for each different ventricle was generated starting from the ventricle reference mask. The obtained masks were then used to calculate the single ventricle volume as described above. Ventricle size was calculated as total µl volume within segmented ventricles and the ventricle volume changes was estimated as a ratio of each day ventricle volume to pre immunization ventricle volume.

Brain tissue volume was manually measured by three independent investigators in 2 animals per group (EAE mice, ventricle normalizing mice and control group), on 2 adjacent slices where it was possible to clearly depict the lateral ventricles and the caudate putamen, the most evident brain regions subjected to volume changes. For each slice, the parenchymal volume was calculated subtracting the volume of the ventricles from the volume of the whole brain area.

### MR Relaxometry

Maps of absolute T_2_ relaxation times were calculated by pixel-wise mono-exponential fitting, implemented in an in-house developed MATLAB program. To quantify the T_2_ relaxation times in the CSF within the ventricular compartment for each obtained map, a mask of the ventricles cleared of the choroid plexus was generated using the FAST tool of FSL. To minimize the influence of partial volume effects generated from neighboring brain tissue, we eroded the automatically generated mask by two voxels. T_2_ relaxation time of the ventricular CSF was calculated as the mean T_2_ relaxation time for all pixels within the mask.

### Data analysis

Three independent blinded investigators were involved in the analysis of the MRI data. All data were thereafter statistically analyzed using GraphPad Prism (GraphPad Software, Inc., La Jolla, CA, USA). The onset of cerebellar lesions was determined by manual inspection. With regards to the onset of ventricle enlargement, a minimum of 10% volume increase from the pre-immunization (baseline) volume was used as threshold. The ventricle normalizing animals were defined as those showing a 10% reduction from the maximum observed ventricular volume. Differences between EAE mice and controls regarding ventricular enlargement and T_2_ relaxation time were analyzed by the student t-test. To compare the onset of cerebellar lesions and ventricular enlargement with the onset of disease we employed a paired t-test since these parameters represent repeated measurements of the same individual mice over time. A p-value of p<0.05 was considered to be statistically significant. A log-rank (Mantel-Cox) test was used to analyze the time-to-event curves and compare the onset of clinical symptoms, cerebellar lesions and ventricle enlargement. 

## Results

### Ventricle enlargement before manifestation of neurological disease

**Figure 1:**
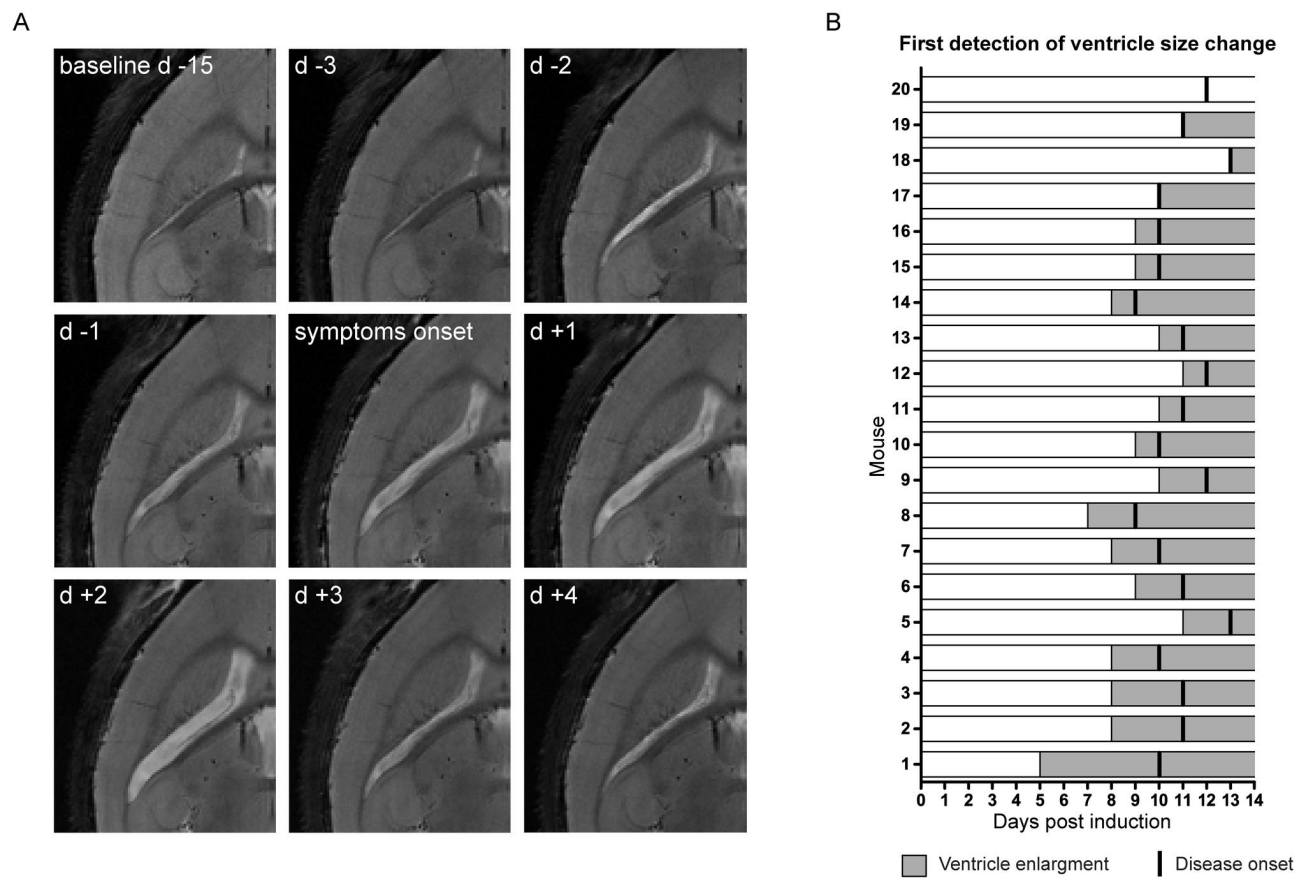
Pre-symptomatic changes in ventricle size in an EAE mouse model. (A) T_2_-weighted horizontal views of the mouse brain show the evolution of changes in ventricle size of a representative mouse from baseline (pre EAE induction) 15 days prior (d-15) to disease manifestation up till 4 days after disease manifestation (d +4). (B) Shown are 20 mice (1–20) exhibiting increase in ventricle volume prior to or concurrent to disease onset. For each animal, gray bars represent the first occurrence of ventricle enlargement and ensuing time points following EAE induction and black vertical lines indicate the symptom onset post immunization. The animals were sorted according to the time difference between first changes in ventricle size (light gray bars) and onset of clinical symptoms post immunization (black vertical lines). Mouse 1 exhibited ventricle enlargement 5 days prior to clinical symptoms, Mouse 17 – Mouse 19 exhibited ventricle enlargement on the same day as clinical symptoms and Mouse 20 showed no ventricle enlargement.

In this study we followed brain modifications during the development of active EAE in a large group of mice (n=20). The first distinct macroscopic change we observed on T_2_-weighted images was an increase in cerebral ventricle size without manifestation of clinical disease (Figure **1A, 1B**). Figure 1A shows a representative mouse (mouse 8 in Figure **1B**) in which ventricle enlargement was detected two days before start of symptoms (d -2). In this EAE mouse we also observed a decrease in ventricle size, three days (d +3) following the symptom onset, defined as the first day of evident motor impairment (tail weakness). Figure 1B summarizes the time-line for all investigated EAE mice and indicates the first day of volume changes in the ventricles in relation to the symptom onset post EAE induction (immunization with CNS antigen).

All immunized mice eventually developed symptomatic EAE. The average day of disease clinical manifestation was 10.8 ± 1.1 (± S.D.) days post immunization, comparable to previous studies [19]. 19 out of 20 mice studied showed an increase in ventricle size and in 16 of these 19 animals, the ventricle enlargement was observed prior to disease manifestation, notably the increase could be detected even up to five days prior to symptom manifestation (mouse 1). In two cases the ventricle enlargement coincided with the symptom onset. On average, EAE mice exhibited an increase in ventricle size 1.6 ±1.2 (± S.D.) days before the first symptom.

### Cerebellar lesion evolution during disease development

**Figure 2:**
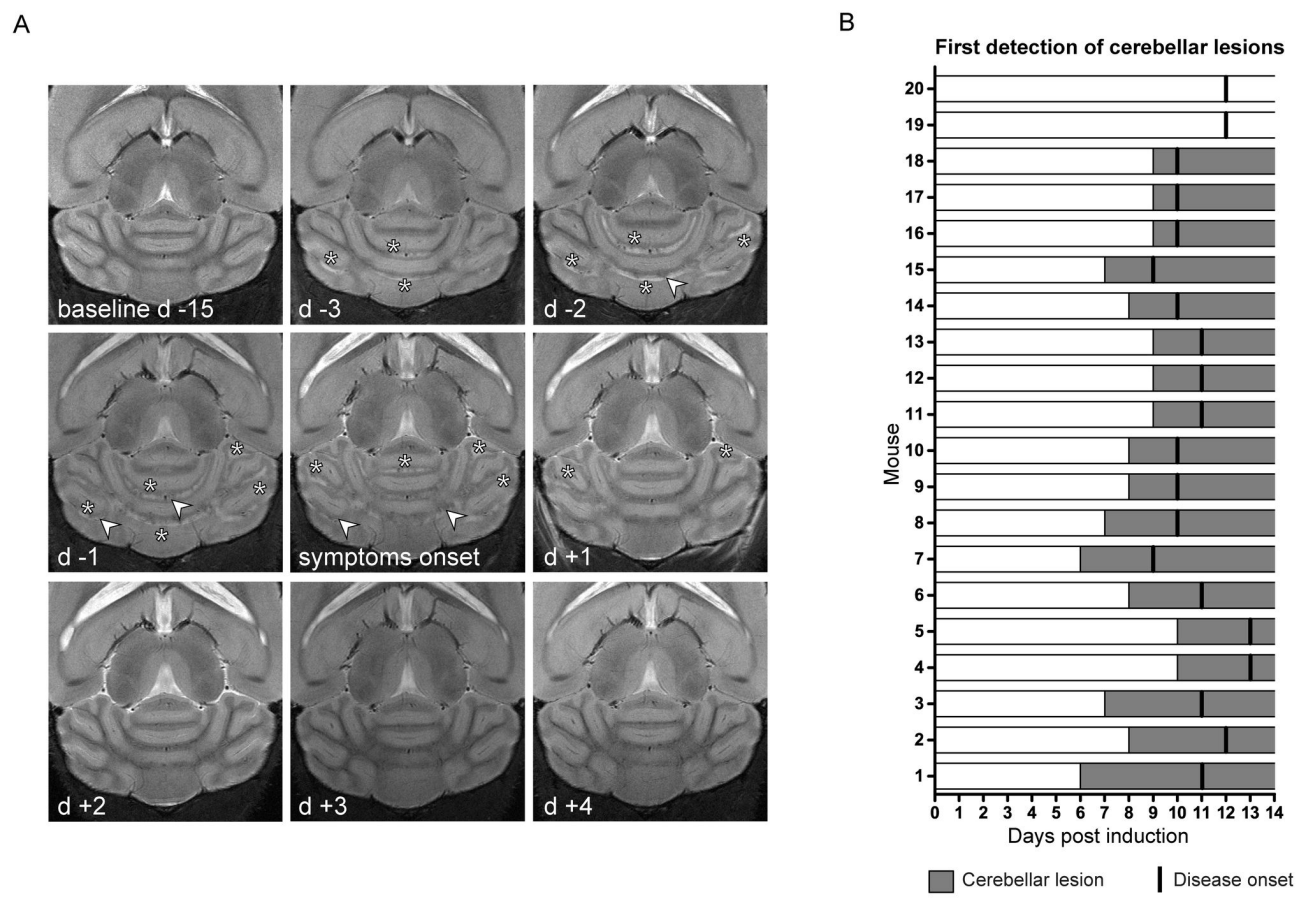
Pre-symptomatic development of cerebellar lesion in an EAE mouse model. (A) T_2_-weighted horizontal views of the mouse cerebellum, which show the temporal progression of lesions in the *arbor vitae* of the cerebellum of a representative mouse starting from pre EAE induction 15 days before clinical symptoms (baseline d-15) up till 2 days after disease manifestation (d +2); stars highlights hyperintense lesion appearance, arrowheads points to the hypointense lesions. (B) Graph showing the first day when cerebellar modifications were observed (gray bars) and the symptom onset (black vertical lines) for all animals in the study. The *x*-axis indicates the time points after EAE induction (0). The animals were sorted according to the time difference between first occurrence in cerebellar lesions and the onset of clinical symptoms post immunization. Mouse 1 exhibited cerebellar lesions 5 days prior to clinical symptoms, Mouse 19 and Mouse 20 showed no cerebellar lesions.

Both hyperintense and hypointense lesions could be detected in multiple brain areas prior to disease manifestation in EAE, however, most commonly hyperintense lesions in the cerebellum [19]. We here investigated the temporal changes of these lesions by T_2_-weighted micro MRI, keeping our main focus on the cerebellum (Figure **2**). Figure 2A shows a representative EAE mouse (mouse 7 in Figure **2B**), in which lesions were identified in the white matter of the cerebellum as hyper-intense regions (Figure **2A**, asterisks) on T_2_-weighted images; these lesions were detected already 3 days before clinical disease onset (d -3). The shape and spatial distribution of these lesions changed over time, also involving other areas of the *arbor vitae* (d -2 and d -1). Some lesions, especially those surrounding small vessels, turned hypo-intense (Figure **2A**, white arrows) and appeared to partially resolve two days after the first neurological symptoms (d +2). Figure 2B recapitulates the temporal differences between the time of first appearance of cerebellar lesions and the time of first symptom manifestation in all EAE mice. Out of 20 animals that developed EAE, 18 showed pre-symptomatic cerebellar lesions on average 2.5 (±1.09 S.D.) days before the first manifestation of clinical signs. Lesion appearance could be observed up to 5 days before disease onset (mouse 1). With the exception of two animals, that did not develop micro MRI visible pathology within the cerebellum, EAE mice commonly manifested early lesions at least one day before appearance of clinical symptoms. Of note, the only mouse that developed clinical disease but no evident changes on MRI (mouse 20) was not only devoid of cerebellar lesions but also exhibited no ventricle enlargement.

The average day of first ventricle size increase was 8.7 ± 2.7 days (± S.D.) post immunization (p.i.) and the average day of first cerebellar lesion occurrence was 7.3 ± 2.8 days (± S.D.) p.i., in comparison to the average day of clinical disease onset, which was 10.8 days p.i. (see above) (Figure **S1A**). We next wanted to determine the fraction of EAE mice undergoing each of these events over the period of time after immunization. We plotted time-to-event curves for first appearance of (i) clinical signs, (ii) ventricular enlargement and (iii) cerebellar lesions against the time span after immunization (Figure **S1B**). We observed that both cerebellar lesions and ventricular enlargement occurred significantly earlier than the appearance of clinical signs (Figure **S1B**); cerebellar lesions vs. clinical signs, *p* < 0.0001; ventricular enlargement vs. clinical signs, *p* = 0.0064.

### Changes in clinical scores are preceded by changes in ventricle size

**Figure 3:**
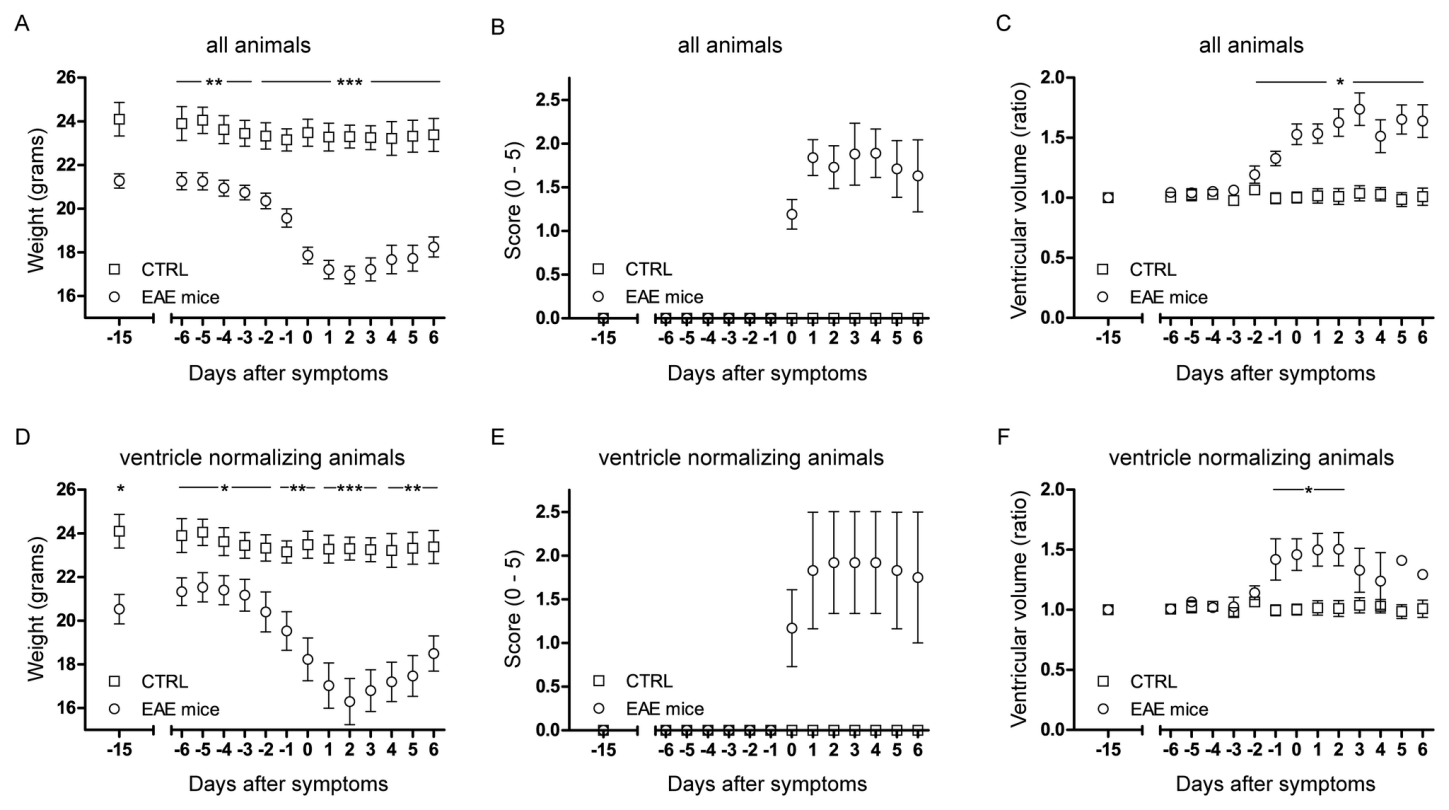
Time-line for weight, score and ventricle volume during EAE development. All the animals were weighed and scored before EAE induction and daily starting from the day of immunization onwards. At baseline and then starting from day 5 after EAE induction micro MRI measurements were performed and the ventricle volumes measured. Graph shows the temporal changes in weight (A), score (B) and ventricle volume (C) for control mice (n=6) and EAE mice (n=20) synchronized to the first day of clinical symptoms. (D) Weight, (E) score and (F) ventricle volume changes of EAE animals that showed remission in ventricle enlargement (n=3) against control animals (n=6).

Next we compared the changes in ventricle volume with EAE signs and symptoms (weight and EAE score) during the progression of disease. Hence, for each mouse, we synchronized all 3 parameters (weight, clinical score and ventricle volume) to the day of first neurological symptoms, defined as time point 0 (Figure **3**). The animal weight started decreasing 3-4 days before disease manifestation and started returning to normal 3-4 days after initiation of symptoms (Figure **3A**). The average score at peak of disease severity was 1.9 ± 0.7 (± S.D.). The maximum was usually reached within the first 3 days following disease manifestation (Figure **3B**).

We normalized the ventricle volumes of each time point to the baseline values (d -15) and plotted the changes in ventricle size over the disease course (Figure **3C**), similar to the weight and score curves. The average mean size of the ventricles at baseline was 10.7 ± 1.1 (± S.D.) mm^3^. In contrast to the non-immunized control mice, the ventricle size of EAE animals started to increase in size on average already two days before clinical symptoms and in some cases expanding 2 times more than baseline volume. For all EAE animals, we compared (i) disease severity and magnitude of ventricular enlargement (Figure **S2A**), (ii) weight loss and magnitude of ventricular enlargement (Figure **S2B**) and (iii) disease severity and onset of ventricular enlargement (Figure **S2C**). However, we did not observe any correlation between the onset or magnitude of ventricular enlargement and the different clinical disease measures.

Notably, in 3 EAE mice (8, 9 and 14 in Figure **1B**), the ventricle size started returning to normal values during disease remission (Figure **3F**). In these EAE mice a reduction in ventricle size started on average 3 days after disease manifestation, at the same time as the peak of symptoms, thus preceding disease remission (Figure **3F**). Although these three mice showed a significant reduction in ventricle size during the remission of the disease (in parallel to the increase in average weight (Figure **3D**), and a decrease in disease score (Figure **3E**), we could not observe any significant differences in the disease scores between these three cases and the rest of the cohort.

Although the present findings occur at an early stage of disease, several days prior to symptom onset, it remains to be excluded that ventricle enlargement occurs as a consequence of brain atrophy, i.e. cell death. Therefore, we followed changes over time in the brain parenchyma volume in mice within the ventricle non-normalizing group and those within the ventricle normalizing group. In comparison to control mice, the group of mice with sustained ventricular enlargement showed a significant decrease in brain volume (Figure **S3A**), interestingly after the time point showing significant enlargement in ventricles (Figure **3C**). In the ventricle normalizing animal group, relative brain parenchymal volumes remained constant. These results suggest that ventricular expansion may be accompanied by compression of the brain parenchyma (Figure **S3B**).

**Figure 4:**
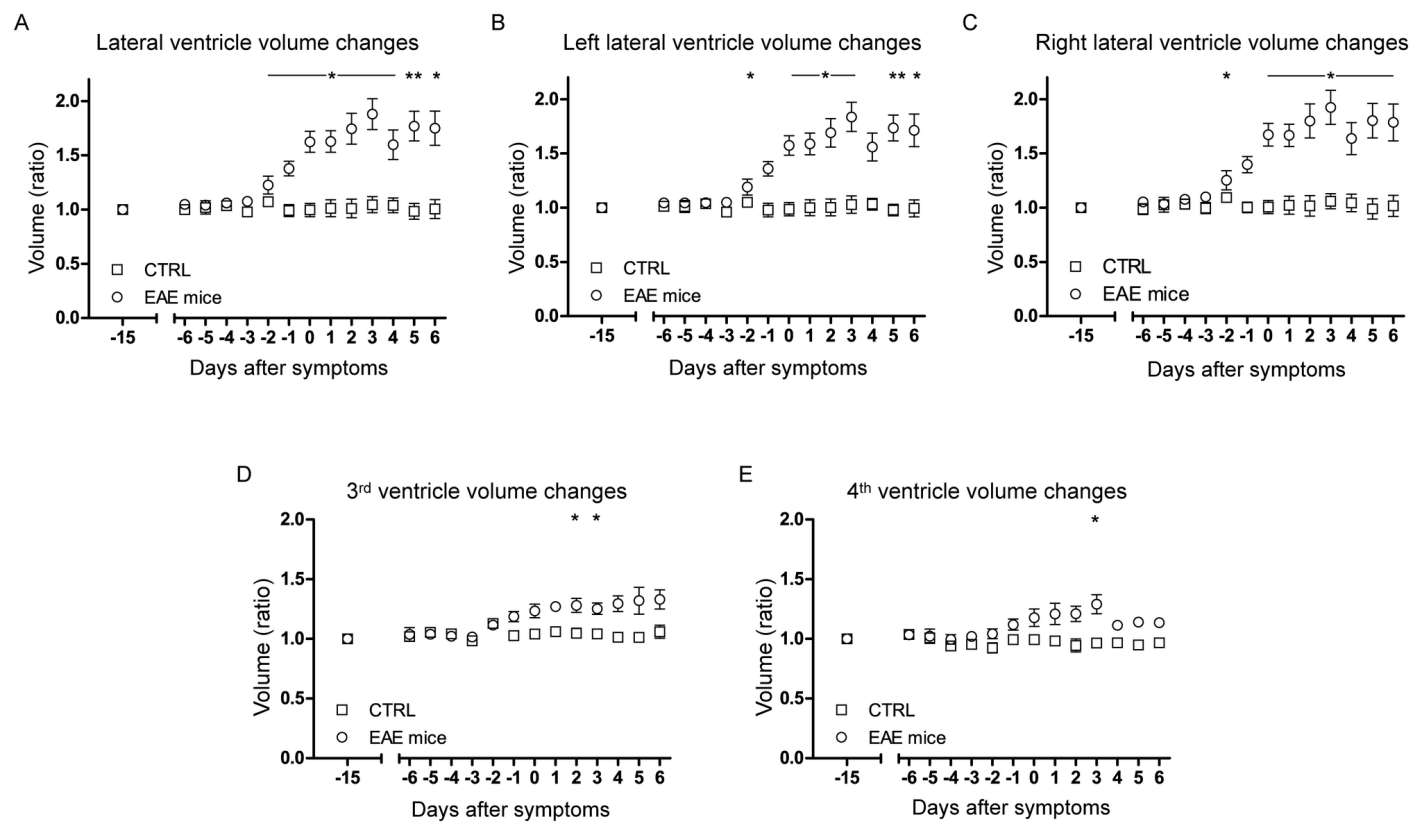
Changes in volume for the single ventricular compartments. Shown is a graphical representation of the temporal changes in volume for both lateral (A), left lateral (B), right lateral (C), third (D) and fourth (E) ventricles for all EAE (n=20) and control (n=6) mice. All points represent a ratio of the daily ventricle volume to the ventricle volume prior to immunization (baseline d-15) and are synchronized to the day of clinical disease onset.

To investigate the possibility that mechanical obstruction between the ventricles (e.g. due to lesion formation) could explain the changes in ventricular volume we calculated the size of each of all four ventricles independently and over time (Figure **4**). All ventricles showed a volume increase before the symptom onset. However, the change in volume in the combined lateral ventricles (Figure **4A**), left ventricle alone (Figure **4B**) and right ventricle alone (Figure **4C**) was much more pronounced than the change in volume in the third (Figure **4D**) and fourth ventricle (Figure **4E**).

In order to exclude the possibility of ventricular changes occurring due to non-specific effects of the adjuvants used during immunization [29], we immunized 6 mice following the same protocol used for the EAE immunization but excluding the PLP peptide. We followed these mice for 10 days after immunization. We could not detect weight loss (Figure **S4A**), disease symptoms (Figure **S4B**), changes in ventricle size (Figure **S4C**) or occurrence of cerebellar lesions in these animals during this time window.

### Ventricle volume increase is related to changes in T_2_ relaxation time of cerebrospinal fluid

**Figure 5:**
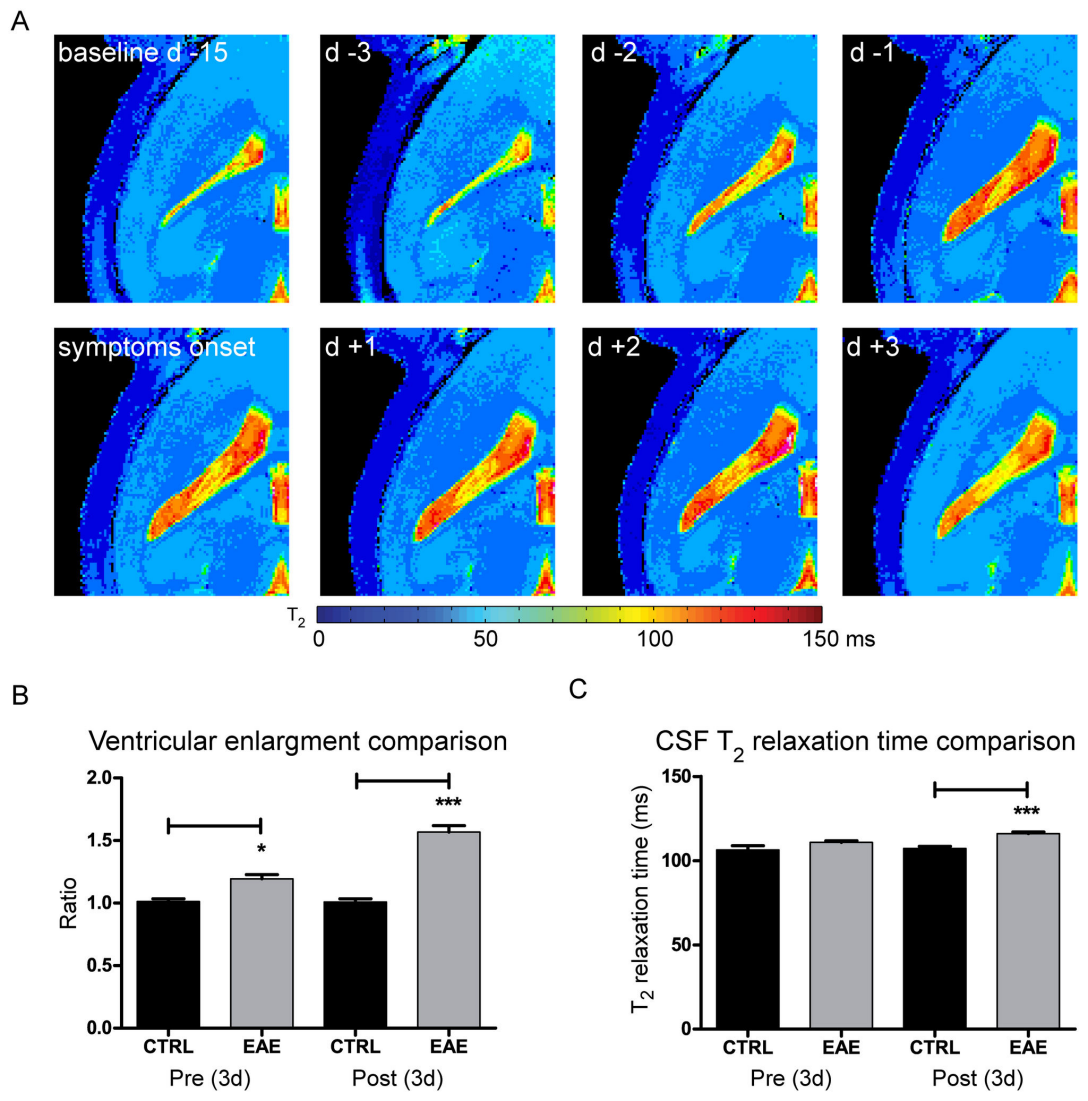
Pre- and post- symptomatic changes in ventricle size and cerebrospinal fluid T_2_ relaxation time. (A) Maps depicting the absolute T_2_ relaxation time (ms) prior to and after disease manifestation. (B) Values for the ventricle volume were grouped into two groups for all EAE animals (3 days before and 3 days after first symptom) and compared with control animals. Shown are the differences in ventricle volume between the control (CTRL) and EAE groups prior to (Pre (3d), *p* = 0.0180) and after (Post (3d), *p* = 0.0003) disease onset. (C) Values for the CSF T_2_ relaxation time were also grouped as above and compared with control animals. Shown are the differences in T_2_ relaxation times between the control (CTRL) and EAE groups prior to (Pre (3d), *p* = 0.0386) and after (Post (3d), *p* = 0.0002) disease manifestation.

To investigate possible changes in water content in the cerebrospinal fluid (CSF), we performed T_2_ relaxometry, which correlates with methods of direct water content quantification in the brain [20,21]. As shown in a representative mouse within the group, a significant increase in T_2_ relaxation time of the CSF also occurred at early stages of the disease and persisted until after disease onset (Figure **5A**). In order to compare the changes in ventricle volume and changes in T_2_ relaxation time, we pooled the data acquired on three consecutive days prior to first clinical symptoms and data acquired during the three days after first clinical symptoms, and compared the changes to age-matched healthy control mice. These control animals were measured in parallel to the EAE mice. When compared to the ventricle size of control animals (volume = 12.7 ± 1.1 (± S.D.) mm^3^), we observed a significant increase in ventricle size in EAE mice prior to clinical disease onset and an even more prominent increase following disease manifestation (Figure **5B**). Also when comparing the CSF T_2_ relaxation times between EAE and control mice, we observed an increase in CSF T_2_ relaxation time both prior to (controls = 106.4 ± 5.83 (± S.D.), EAE mice = 110.8 ± 4.18 (± S.D.), *p* = 0.0586) and following (controls = 107.3 ± 3.10 (± S.D.), EAE mice = 116 ± 4.29 (± S.D.), *p* = 0.0004) disease onset (Figure **5C**).

## Discussion

Studying the early processes during CNS inflammation is important to gain a better insight into how immune cell invasion may affect the autoimmune response. In this study we identified an early ventricular enlargement prior to cerebral disease manifestation in a mouse model of encephalomyelitis.

In the animal model, the increase in ventricular size was accompanied by increased free water content in CSF as demonstrated by increased T_2_ relaxation times in the CSF compartment. In some EAE mice we observed a contraction in ventricle size at the time between disease peak and prior to remission, indicating a possible reversibility in the factor contributing to enlargement of the ventricles. Likely as a consequence to ventricular enlargement, in the group of animals with sustained ventricular enlargement we observed a decrease in brain parenchyma volume. In contrast, this finding did not hold true in the ventricle normalizing animals. In the non-normalizing animals the brain volume reduction occurred after ventricle enlargement. Thus, we may exclude brain atrophy as primary source of ventricular enlargement in our study – a finding further supported by the very early time point ventricular enlargement is detectable. Prior to the macroscopic changes in the ventricles, we also detected microscopic alterations in the brain parenchyma. Microscopic changes involved evolving hyper- and hypo-intense regions in T_2_-weighted images, particularly in the cerebellar white matter and as early as three days before symptom onset. The latter is in line with our first micro MRI study in EAE mice where we corroborated using immunohistology the presence of hypo-intense lesions in T_2_-weighted images to areas of immune cell infiltration [19].

One possible explanation for the ventricular enlargement could be an impediment in cerebral CSF flow resulting from a physical obstruction at the interventricular foramen (Monro), such as in obstructive hydrocephalus. We therefore measured the changes in volume in all four ventricles to determine whether the ventricles downstream of the lateral ventricles also changed in size. The analysis of each separate ventricle revealed that each ventricular compartment increased in size prior to clinical symptom onset. Also a few days before disease manifestation, we detected an increase in CSF water content within the ventricles as reflected from the T_2_ relaxation time. Several previous reports have demonstrated a linear correlation between spin-spin relaxation time (T_2_) measurements and *ex vivo* desiccation methods to quantify tissue water content in brain [20,21] and other tissue [30]. As reference, *in vitro* modifications in water content from 70% to 85% were reported to change T_2_ relaxation time from 100 ms to 200 ms [20].

Our results appear to preclude the hypothesis that volume increase in the ventricles could be the result of an obstruction in one of the foramina connecting the ventricles. These results rather suggest that the increase in ventricular size could be either the result of a dysregulation in CSF homeostasis (e.g. a deficit in CSF reabsorption or an increase in CSF production). Alternatively, a disruption of the tight junctions (TJ) of the BBB and blood CSF barrier may increase the flux of solutes through the barrier leading to fluid leakage into the CSF-filled compartments. In this perspective it has been shown that an alteration of BBB TJ proteins and changes in microcirculation, associated with the presence of inflammatory cells, occur during EAE and MS [13,31–33]. On the other hand, we recently reported a contrast agent-enriched ventricular system in EAE suggesting a leakage into the ventricles possibly via the blood CSF barrier during disease [19]. Using a flow compensated gradient echo technique, we observed an outflow of Gd-DTPA into the ventricles of EAE but not healthy animals [19]. Either of these possibilities suggests that ventricle size may serve as a potential indicator of the level of inflammation in the CNS, even prior to other more conventional measures (e.g. contrast enhancing lesions).

Several CNS routes exist for CSF drainage that might be involved in the communication with the lymphatic system. The relevance of immune cell trafficking between the lymphatic system and CNS has been described in models of spontaneous EAE [34]. Important communication routes include anatomical sites (such as the cribriform plate) associated with lymphatics (eg. those associated with the olfactory or other cranial nerves) [35]. Immune cells, including memory CD4+ T cells, follow these communication routes during autoimmune neuroinflammation [2]. More recently, it was also shown that leukocytes infiltrate periventricular tissue as well as perivascular spaces prior to clinical symptom onset [36]. In agreement with this study, we have also recently shown an extensive accumulation of inflammatory cells on the ventral side of the forebrain, midbrain and brainstem as shown by fluorine/proton MRI [37].

In all of our EAE cases, the fluctuations in ventricular volume were relatively fast and cannot be explained by brain atrophy that is a slow and gradual process occurring over several years [38]. Furthermore, in a recent study with MS patients that were followed up MRI for a period of 5 years after first diagnosis, the percentage ventricular volume change was found to be higher during the first 2 years compared to the ensuing 3 years [39]. The rate of early ventricular enlargement was also suggested in this study to be more predictive of disease progression than lesion measures or whole brain atrophy rate [39].

In summary, our present findings in the EAE model provide first indications that the progression of encephalomyelitis is accompanied by substantial fluctuations in ventricle size already in the earliest stages of disease. Several mechanisms could be involved in this observation, including a disruption in CSF homeostasis or fluctuations in the edematous milieu of inflammatory cells and their products. Further preclinical and clinical investigations are warranted to elucidate the basis leading to ventricular enlargement and to explore the mechanisms and causative factors that could precipitate these macroscopic changes during encephalomyelitis.

## Supporting Information

Figure S1 **Overview of the pre-symptomatic brain alterations.**
(A) Shown are, for each animal, the first day of cerebellar lesion appearance (*p* < 0.0001) and the first day of ventricle enlargement (*p* < 0.0001) are shown, compared to the days of symptom onset (n= 20). (B) Time-to-event curves to compare the first occurrence of ventricle enlargement and cerebellar lesions relative to clinical onset. Statistical significance: cerebellar lesions vs. clinical signs, *p* < 0.0001; ventricular enlargement vs. clinical signs, *p* = 0.0064.
(TIF)

Figure S2 **No relation between magnitude or occurrence of ventricle enlargement and clinical disease measures.**
(A) Comparison between disease severity and magnitude of ventricular enlargement. (B) Comparison between weight loss and magnitude of ventricular enlargement. (C) Comparison between disease severity and occurrence of ventricular enlargement.
(TIF)

Figure S3 **Time-line of changes in brain parenchyma volume during EAE development.**
Brain parenchyma was measured in slices depicting the caudate putamen and lateral ventricles. The parenchymal volumes for all animals were centered on day of symptom onset. Temporal changes in the parenchymal volume of mice with sustained ventricle enlargement (A) and parenchymal volume of mice with normalizing ventricles (B) were compared to controls.
(TIF)

Figure S4 **Timeline of weight and ventricle size and score of sham immunized animals.**
All the animals were weighed and scored before sham immunization and daily thereafter. Micro MRI measurements were performed at baseline and then from day 5 to day 10 after sham immunization and the ventricle volumes measured. Temporal changes in weight (A), score (B) and ventricle volume (C) are shown (n=6).
(TIF)

## References

[1] Sallusto F, Impellizzieri D, Basso C, Laroni A, Uccelli A et al. (2012) T-cell trafficking in the central nervous system. Immunol Rev 248: 216-227. doi:10.1111/j.1600-065X.2012.01140.x. PubMed: 22725964. 22725964 10.1111/j.1600-065X.2012.01140.x

[2] Giunti D, Borsellino G, Benelli R, Marchese M, Capello E et al. (2003) Phenotypic and functional analysis of T cells homing into the CSF of subjects with inflammatory diseases of the CNS. J Leukoc Biol 73: 584-590. doi:10.1189/jlb.1202598. PubMed: 12714572. 12714572 10.1189/jlb.1202598

[3] Ruiz-Cabello J, Barnett BP, Bottomley PA, Bulte JW (2011) Fluorine (19F) MRS and MRI in biomedicine. NMR Biomed 24: 114-129. doi:10.1002/nbm.1570. PubMed: 20842758. 20842758 10.1002/nbm.1570 PMC3051284

[4] Ousman SS, Kubes P (2012) Immune surveillance in the central nervous system. Nat Neurosci 15: 1096-1101. doi:10.1038/nn.3161. PubMed: 22837040. 22837040 10.1038/nn.3161 PMC7097282

[5] Kivisäkk P, Mahad DJ, Callahan MK, Trebst C, Tucky B et al. (2003) Human cerebrospinal fluid central memory CD4+ T cells: evidence for trafficking through choroid plexus and meninges via P-selectin. Proc Natl Acad Sci U S A 100: 8389-8394. doi:10.1073/pnas.1433000100. PubMed: 12829791. 12829791 10.1073/pnas.1433000100 PMC166239

[6] Wilson EH, Weninger W, Hunter CA (2010) Trafficking of immune cells in the central nervous system. J Clin Invest 120: 1368-1379. doi:10.1172/JCI41911. PubMed: 20440079. 20440079 10.1172/JCI41911 PMC2860945

[7] Greter M, Heppner FL, Lemos MP, Odermatt BM, Goebels N et al. (2005) Dendritic cells permit immune invasion of the CNS in an animal model of multiple sclerosis. Nat Med 11: 328-334. doi:10.1038/nm1197. PubMed: 15735653. 15735653 10.1038/nm1197

[8] Kivisäkk P, Imitola J, Rasmussen S, Elyaman W, Zhu B et al. (2009) Localizing central nervous system immune surveillance: meningeal antigen-presenting cells activate T cells during experimental autoimmune encephalomyelitis. Ann Neurol 65: 457-469. doi:10.1002/ana.21379. PubMed: 18496841. 18496841 10.1002/ana.21379 PMC3305810

[9] Kerfoot SM, Kubes P (2002) Overlapping roles of P-selectin and alpha 4 integrin to recruit leukocytes to the central nervous system in experimental autoimmune encephalomyelitis. J Immunol 169: 1000-1006. PubMed: 12097407. 12097407 10.4049/jimmunol.169.2.1000

[10] Piccio L, Rossi B, Scarpini E, Laudanna C, Giagulli C et al. (2002) Molecular mechanisms involved in lymphocyte recruitment in inflamed brain microvessels: critical roles for P-selectin glycoprotein ligand-1 and heterotrimeric G(i)-linked receptors. J Immunol 168: 1940-1949. PubMed: 11823530. 11823530 10.4049/jimmunol.168.4.1940

[11] Engelhardt B, Coisne C (2011) Fluids and barriers of the CNS establish immune privilege by confining immune surveillance to a two-walled castle moat surrounding the CNS castle. Fluids Barriers CNs 8: 4. doi:10.1186/2045-8118-8-4. PubMed: 21349152. 21349152 10.1186/2045-8118-8-4 PMC3039833

[12] Dawson JW (1916). XVIII—Histol Disseminated Sclerosis Transactions R Soc Edinb 50: 517-740. doi:10.1017/S0080456800027174.

[13] Plumb J, McQuaid S, Mirakhur M, Kirk J (2002) Abnormal endothelial tight junctions in active lesions and normal-appearing white matter in multiple sclerosis. Brain Pathol 12: 154-169. PubMed: 11958369. 11958369 10.1111/j.1750-3639.2002.tb00430.x PMC8095734

[14] Achiron A, Faibel M (2002) Sandlike appearance of Virchow-Robin spaces in early multiple sclerosis: a novel neuroradiologic marker. AJNR Am J Neuroradiol 23: 376-380. PubMed: 11901003. 11901003 PMC7975312

[15] Wuerfel J, Haertle M, Waiczies H, Tysiak E, Bechmann I et al. (2008) Perivascular spaces--MRI marker of inflammatory activity in the brain? Brain 131: 2332-2340. doi:10.1093/brain/awn171. PubMed: 18676439. 18676439 10.1093/brain/awn171

[16] Nouls JC, Izenson MG, Greeley HP, Johnson GA (2008) Design of a superconducting volume coil for magnetic resonance microscopy of the mouse brain. J Magn Reson 191: 231-238. doi:10.1016/j.jmr.2007.12.018. PubMed: 18221901. 18221901 10.1016/j.jmr.2007.12.018 PMC2361158

[17] Baltes C, Radzwill N, Bosshard S, Marek D, Rudin M (2009) Micro MRI of the mouse brain using a novel 400 MHz cryogenic quadrature RF probe. NMR Biomed 22: 834-842. doi:10.1002/nbm.1396. PubMed: 19536757. 19536757 10.1002/nbm.1396

[18] Wagenhaus B, Pohlmann A, Dieringer MA, Els A, Waiczies H et al. (2012) Functional and morphological cardiac magnetic resonance imaging of mice using a cryogenic quadrature radiofrequency coil. PLOS ONE 7: e42383. doi:10.1371/journal.pone.0042383. PubMed: 22870323. 22870323 10.1371/journal.pone.0042383 PMC3411643

[19] Waiczies H, Millward JM, Lepore S, Infante-Duarte C, Pohlmann A et al. (2012) Identification of Cellular Infiltrates during Early Stages of Brain Inflammation with Magnetic Resonance Microscopy. PLOS ONE 7: e32796. doi:10.1371/journal.pone.0032796. PubMed: 22427887. 22427887 10.1371/journal.pone.0032796 PMC3299701

[20] Kamman RL, Go KG, Brouwer W, Berendsen HJ (1988) Nuclear magnetic resonance relaxation in experimental brain edema: effects of water concentration, protein concentration, and temperature. Magn Reson Med 6: 265-274. doi:10.1002/mrm.1910060304. PubMed: 3362061. 3362061 10.1002/mrm.1910060304

[21] Qiao M, Malisza KL, Del Bigio MR, Tuor UI (2001) Correlation of cerebral hypoxic-ischemic T2 changes with tissue alterations in water content and protein extravasation. Stroke 32: 958-963 10.1161/01.str.32.4.958 11283397

[22] Aktas O, Waiczies S, Smorodchenko A, Dorr J, Seeger B et al. (2003) Treatment of relapsing paralysis in experimental encephalomyelitis by targeting Th1 cells through atorvastatin. J Exp Med 197: 725-733. doi:10.1084/jem.20021425. PubMed: 12629065. 12629065 10.1084/jem.20021425 PMC2193848

[23] Smith SM, Jenkinson M, Woolrich MW, Beckmann CF, Behrens TE et al. (2004) Advances in functional and structural MR image analysis and implementation as FSL. NeuroImage 23 Suppl 1: S208-S219. doi:10.1016/j.neuroimage.2004.07.051. PubMed: 15501092. 15501092 10.1016/j.neuroimage.2004.07.051

[24] Woolrich MW, Jbabdi S, Patenaude B, Chappell M, Makni S et al. (2009) Bayesian analysis of neuroimaging data in FSL. Neuroimage 45: S173-S186. doi:10.1016/j.neuroimage.2008.10.055. PubMed: 19059349. 19059349 10.1016/j.neuroimage.2008.10.055

[25] Jenkinson M, Beckmann CF, Behrens TE, Woolrich MW, Smith SM (2012) Fsl. NeuroImage 62: 782-790. doi:10.1016/j.neuroimage.2011.09.015. PubMed: 21979382. 21979382 10.1016/j.neuroimage.2011.09.015

[26] Jenkinson M, Pechaud M, Smith S (2005) BET2: MR-based estimation of brain, skull and scalp surfaces. Eleventh Annual Meeting of the Organization for Human Brain Mapping.

[27] Zhang Y, Brady M, Smith S (2001) Segmentation of brain MR images through a hidden Markov random field model and the expectation-maximization algorithm. IEEE Trans Med Imaging 20: 45-57. doi:10.1109/42.906424. PubMed: 11293691. 11293691 10.1109/42.906424

[28] Jenkinson M, Bannister P, Brady M, Smith S (2002) Improved optimization for the robust and accurate linear registration and motion correction of brain images. NeuroImage 17: 825-841. doi:10.1006/nimg.2002.1132. PubMed: 12377157. 12377157 10.1016/s1053-8119(02)91132-8

[29] Rivest S (2003) Molecular insights on the cerebral innate immune system. Brain Behav Immun 17: 13-19. doi:10.1016/S0889-1591(02)00055-7. PubMed: 12615045. 12615045 10.1016/s0889-1591(02)00055-7

[30] Lüsse S, Claassen H, Gehrke T, Hassenpflug J, Schünke M et al. (2000) Evaluation of water content by spatially resolved transverse relaxation times of human articular cartilage. Magn Reson Imaging 18: 423-430. doi:10.1016/S0730-725X(99)00144-7. PubMed: 10788720. 10788720 10.1016/s0730-725x(99)00144-7

[31] Wolburg H, Wolburg-Buchholz K, Kraus J, Rascher-Eggstein G, Liebner S et al. (2003) Localization of claudin-3 in tight junctions of the blood-brain barrier is selectively lost during experimental autoimmune encephalomyelitis and human glioblastoma multiforme. Acta Neuropathol 105: 586-592. PubMed: 12734665. 12734665 10.1007/s00401-003-0688-z

[32] Morgan L, Shah B, Rivers LE, Barden L, Groom AJ et al. (2007) Inflammation and dephosphorylation of the tight junction protein occludin in an experimental model of multiple sclerosis. Neuroscience 147: 664-673. doi:10.1016/j.neuroscience.2007.04.051. PubMed: 17560040. 17560040 10.1016/j.neuroscience.2007.04.051

[33] Wuerfel J, Paul F, Zipp F (2007) Cerebral blood perfusion changes in multiple sclerosis. J Neurol Sci 259: 16-20. doi:10.1016/j.jns.2007.02.011. PubMed: 17382348. 17382348 10.1016/j.jns.2007.02.011

[34] Furtado GC, Marcondes MC, Latkowski JA, Tsai J, Wensky A et al. (2008) Swift entry of myelin-specific T lymphocytes into the central nervous system in spontaneous autoimmune encephalomyelitis. J Immunol 181: 4648-4655. PubMed: 18802067. 18802067 10.4049/jimmunol.181.7.4648 PMC3973185

[35] Ransohoff RM, Kivisäkk P, Kidd G (2003) Three or more routes for leukocyte migration into the central nervous system. Nat Rev Immunol 3: 569-581. doi:10.1038/nri1130. PubMed: 12876559. 12876559 10.1038/nri1130

[36] Schmitt C, Strazielle N, Ghersi-Egea JF (2012) Brain leukocyte infiltration initiated by peripheral inflammation or experimental autoimmune encephalomyelitis occurs through pathways connected to the CSF-filled compartments of the forebrain and midbrain. J Neuroinflammation 9: 187. doi:10.1186/1742-2094-9-187. PubMed: 22870891. 22870891 10.1186/1742-2094-9-187 PMC3458946

[37] Waiczies H, Lepore S, Drechsler S, Qadri F, Purfurst B et al. (2013) Visualizing brain inflammation with a shingled-leg radio-frequency head probe for 19F/1H MRI. Sci Rep. p. 3: 1280 10.1038/srep01280 PMC3573344 23412352

[38] Miller DH, Barkhof F, Frank JA, Parker GJ, Thompson AJ (2002) Measurement of atrophy in multiple sclerosis: pathological basis, methodological aspects and clinical relevance. Brain 125: 1676-1695. doi:10.1093/brain/awf177. PubMed: 12135961. 12135961 10.1093/brain/awf177

[39] Lukas C, Minneboo A, de Groot V, Moraal B, Knol DL et al. (2010) Early central atrophy rate predicts 5 year clinical outcome in multiple sclerosis. J Neurol Neurosurg, Psychiatry 81: 1351-1356. doi:10.1136/jnnp.2009.199968. PubMed: 20826873. 20826873 10.1136/jnnp.2009.199968

