# Supplementary figures and images for: Enlargement of Cerebral Ventricles as an Early Indicator of Encephalomyelitis
Source: PLoS One. 2013 Aug 22;8(8):e72841. doi: 10.1371/journal.pone.0072841 (PMC3750011; doi:10.1371/journal.pone.0072841)

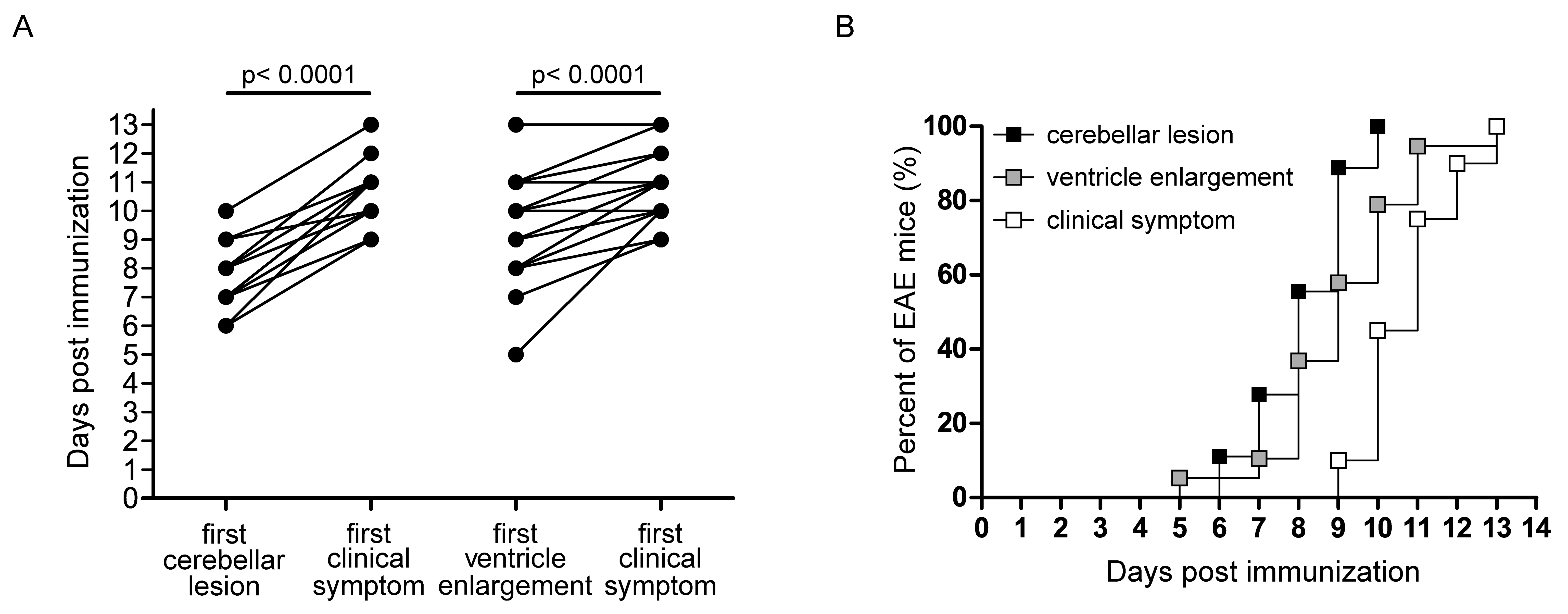

Supplement: Figure S1 — Overview of the pre-symptomatic brain alterations. (A) Shown are, for each animal, the first day of cerebellar lesion appearance (p < 0.0001) and the first day of ventricle enlargement (p < 0.0001) are shown, compared to the days of symptom onset (n= 20). (B) Time-to-event curves to compare the first occurrence of ventricle enlargement and cerebellar lesions relative to clinical onset. Statistical significance: cerebellar lesions vs. clinical signs, p < 0.0001; ventricular enlargement vs. clinical signs, p = 0.0064. (TIF) [file pone.0072841.s001.tif]

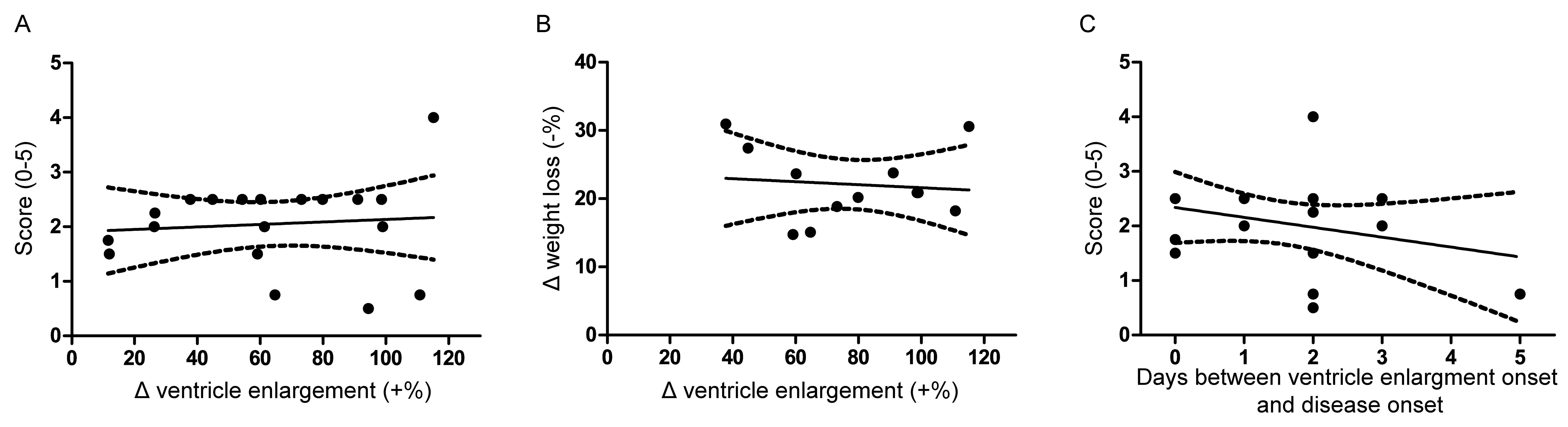

Supplement: Figure S2 — No relation between magnitude or occurrence of ventricle enlargement and clinical disease measures. (A) Comparison between disease severity and magnitude of ventricular enlargement. (B) Comparison between weight loss and magnitude of ventricular enlargement. (C) Comparison between disease severity and occurrence of ventricular enlargement. (TIF) [file pone.0072841.s002.tif]

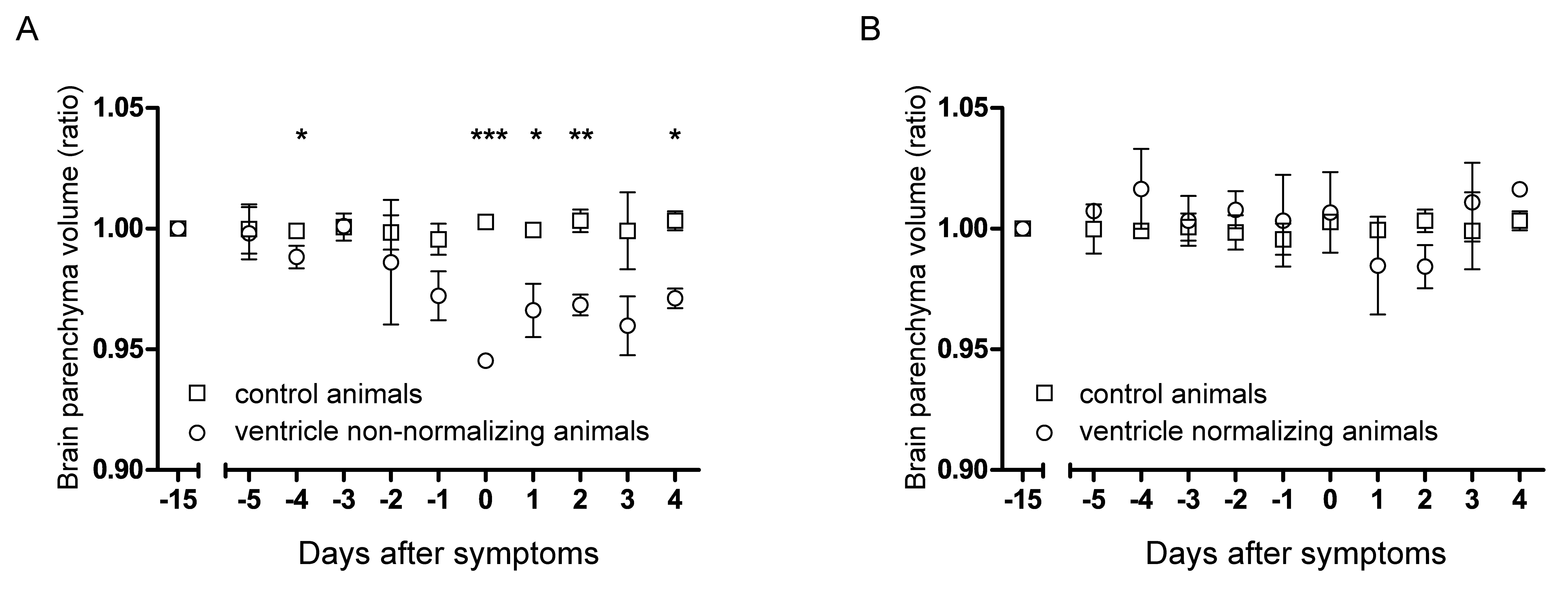

Supplement: Figure S3 — Time-line of changes in brain parenchyma volume during EAE development. Brain parenchyma was measured in slices depicting the caudate putamen and lateral ventricles. The parenchymal volumes for all animals were centered on day of symptom onset. Temporal changes in the parenchymal volume of mice with sustained ventricle enlargement (A) and parenchymal volume of mice with normalizing ventricles (B) were compared to controls. (TIF) [file pone.0072841.s003.tif]

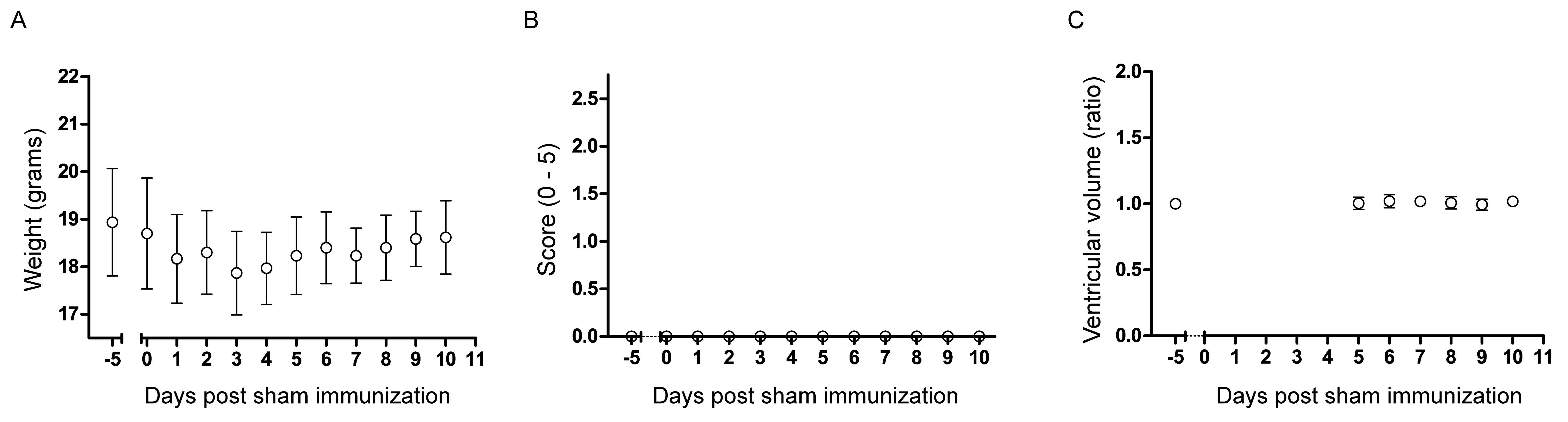

Supplement: Figure S4 — Timeline of weight and ventricle size and score of sham immunized animals. All the animals were weighed and scored before sham immunization and daily thereafter. Micro MRI measurements were performed at baseline and then from day 5 to day 10 after sham immunization and the ventricle volumes measured. Temporal changes in weight (A), score (B) and ventricle volume (C) are shown (n=6). (TIF) [file pone.0072841.s004.tif]
